# Structural basis for promiscuity in ligand recognition by *yjdF* riboswitch

**DOI:** 10.1038/s41421-024-00663-2

**Published:** 2024-04-02

**Authors:** Daniel Krochmal, Christina Roman, Anna Lewicka, Yaming Shao, Joseph A. Piccirilli

**Affiliations:** 1 Department of Biochemistry and Molecular Biology, University of Chicago, Chicago, IL USA; 2 Department of Chemistry, University of Chicago, Chicago, IL USA

**Keywords:** X-ray crystallography, Riboswitches

Dear Editor,

The *yjdF* motif RNA is a riboswitch found primarily in Firmicutes, located typically in the 5’UTR of the orphan YjdF protein gene. This riboswitch operates as a translational “ON” switch, upregulating translation upon ligand binding either by suppressing a repressive interaction with the ribosome-binding site (RBS) or by mimicking tRNA^1,2^. While neither the function of YjdF protein nor the natural ligand of the *yjdF* riboswitch is known, the *yjdF* riboswitch has captured interest due to its capacity to bind to a wide range of multiring, planar, azaaromatic compounds, that draws functional parallels to PadR proteins, often encoded near the *yjdF* gene when the riboswitch is absent^3,4^. This binding promiscuity makes the *yjdF* riboswitch stand out among riboswitches, as most typically recognize their ligands with high selectivity. Studies implicate the presence of a ligand binding pocket with 1:1 binding stoichiometry^2,3^; however, the structural basis of this promiscuity remains unclear.

To reveal the architecture of the *yjdF* aptamer domain, we solved crystal structures of the *yjdF* riboswitch from *Ruminococcus gauvreauii* with chelerythrine and proflavine, both binding the *yjdF* aptamer domain with low nanomolar affinity and activating the expression of downstream genes^2,3^ (Fig. 1a; Supplementary Table S1). We employed antibody-assisted RNA crystallography by modifying the non-conserved P2a loop for Fab BL3-6 binding and utilized potassium osmate(VI) soaking to further assist in phasing^3,5^ (Fig. 1b, d). The crystals diffracted to 2.77 Å and 3.05 Å resolution for chelerythrine and proflavine, respectively. Both crystals belonged to the P1 space group and contained two complexes per asymmetric unit.

**Fig. 1:**
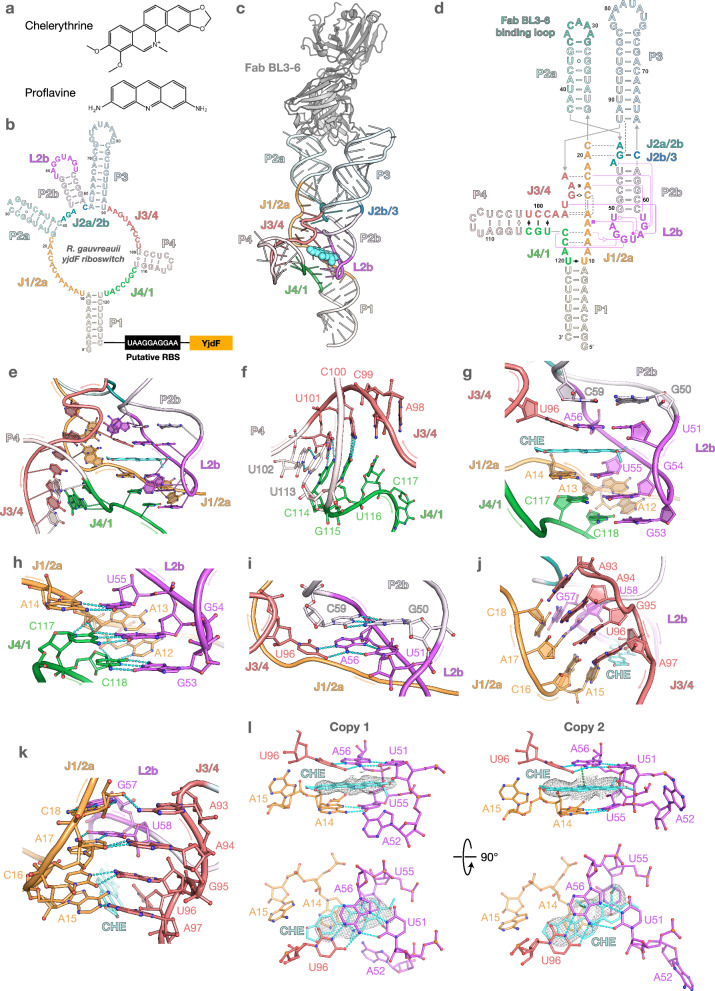
Crystal structure of *yjdF* riboswitch in complex with chelerythrine. **a** Chemical structure of the azaaromatic compounds co-crystallized with the *yjdF* riboswitch. **b** Predicted secondary structure of the *yjdF* riboswitch^1,3^. **c** Crystal structure of *yjdF*/chelerythrine complex co-crystallized with Fab BL3-6. **d** Secondary structure of *yjdF* riboswitch derived from the crystal structure. Interactions are denoted using the Westhof-Leontis notation^9^, with the L2b-mediated interactions colored magenta. Dashed lines indicate interactions mediated by 2’OH. **e** Central core of *yjdF* riboswitch with the ligand-binding site. **f** Interactions between J3/4 and J4/1 at the base of P4 stem that sequester the RBS-complementary motif. **g** Overall arrangement of the nucleotides forming the top and the bottom of the ligand-binding pocket. A52 is omitted for clarity. Detailed interactions forming the bottom (**h**) and top (**i**) of the ligand-binding pocket. **j**, **k** Overall arrangement (**j**) and detailed interactions (**k**) forming the side of the binding pocket. **l** Specific interactions in the ligand-binding site from both copies of *yjdF* complex in the asymmetric unit. Cyan dashes indicate heteroatoms within hydrogen-bonding distance (3.5 Å). Green dashes indicate interactions mediated by the ligand. Meshes represent the composite omit map at 1.5σ and carve radius 1.8 Å.

Previous secondary structure predictions and in-line probing suggest that the *yjdF* aptamer domain from *R. gauvreauii* contains 5 stem-loops, linked by five single-stranded, joining regions^3^ (Fig. 1b). Our structures corroborate these observations but reveal extensive interactions involving the joining regions that contribute to the architecture of the ligand-binding pocket. Our structures show that both the chelerythrine- and proflavine-bound *yjdF* riboswitch adopt the same global architecture (RMSD 1.528 Å; Supplementary Figs. S1, S2). Four stems, P1, P2a, P3 and P4, protrude radially from the central core, while P2b, stacks coaxially with P3 and uses its loop (L2b) to form interactions with J1/2a, J4/1 and J3/4 that create the ligand binding pocket (Fig. 1c, d; Supplementary Fig. S3).

The P4 stem contains a UUCCUCCUUA motif (residues 101–110) complementary to the RBS located downstream from the aptamer domain, consistent with a potential role in the riboswitch regulatory mechanism^1,3,6^. The P1, P2a, P2b and P3 stems lie on a common plane, while P4 projects away from this plane at a right angle. The P4 stem consists of four canonical base-pairs supported by non-canonical interactions between nucleotides of J3/4 and J4/1 near the base of the stem and in proximity to the ligand-binding site (Fig. 1d–f). The most proximal pair involves C99 and U116, which reside within the P4 helical stack directing their WCF faces towards one another, possibly forming a single hydrogen bond (H-bond, 3.9 Å) (Fig. 1d–f; Supplementary Fig. S4). This architecture could make P4 stability sensitive to ligand binding and therefore prone to unwinding in the absence of ligand, which would free the RBS-complementary sequence for downstream hybridization. Consistent with conformational flexibility in this region, the two molecules in the asymmetric unit exhibit structural differences (Supplementary Figs. S2, S11 and Note S1). In the second copy, the putative hydrogen bonding heteroatoms of C99 and U116 are further apart (5.4 Å), and the P4 helix extends from the central core at a sharper angle (Supplementary Figs. S2, S4).

The ligand-binding pocket, which is the same in chelerythrine- and proflavine-bound structures (RMSD 1.129 Å; Supplementary Figs. S5, S6), resides inside the central core of the *yjdF* aptamer domain. The P2b stem-loop anchors the core via a T-loop-like fold^7^, with the joining regions J4/1, J3/4, and J1/2a assembled around its minor groove face (Fig. 1c, g; Supplementary Fig. S7). The base of the pocket is created via G53 and G54 from L2b forming WCF base pairs with C118 and C117 from J4/1, further stabilized by a ribose zipper (Fig. 1g, h; Supplementary Fig. S7). A WCF-Hoogsteen base pair between U55 and A14 from L2b and J1/2a, respectively, stacks on top of the G53-G54/C118-C117 base pairs and serves as the platform for stacking with the bottom face of the ligand (Fig. 1h; Supplementary Figs. S7, S8). Together with the ribose zipper, these three base pairs create a stable platform for the ligand (Fig. 1g). The importance of these interactions for riboswitch function is highlighted by the high conservation of these positions in this riboswitch class^3^ (Supplementary Fig. S1).

The top of the ligand binding pocket is formed by the apical part of L2b and is supported by internal canonical base pairing of the P2b stem (Fig. 1g, i; Supplementary Fig. S7). The platform for stacking with the top face of the ligand is formed by a base triple involving highly conserved nucleotides: A56 from L2b utilizes its WCF face to pair with U96 from J3/4 and its Hoogsteen face to interact with U51 from L2b^3^ (Fig. 1i; Supplementary Figs. S7, S8). The top and bottom ligand stacking platforms run parallel to one another, forming a pocket capable of sandwiching planar aromatic compounds (Fig. 1g).

Interactions of J1/2a with J3/4 and nucleotides at the 3’-end of L2b enclose the ligand from the side (Fig. 1j; Supplementary Fig. S7). Specifically, the A15-C18 nucleotides of J1/2a and A93, A94, G95, and A97 of J3/4 form anti-parallel base stacks perpendicular to the ligand plane with A15 forming the innermost layer, anchored by an H-bond to A97 and stacking with the C16-G95 trans-WCF base pair (Fig. 1j, k; Supplementary Fig. S7). These residues are highly conserved within the *yjdF* riboswitch family, underscoring the critical role of these interactions in the function of this riboswitch^3^ (Supplementary Fig. S1). Further supporting the architecture of the ligand binding pocket are two base triples involving L2b: the A17-U58 WCF base pair and A94, which interacts with the sugar edge of A17 and stacks upon G95; and C18-G57 with A93 hydrogen bonding to 2’OH of C18 (Fig. 1k; Supplementary Fig. S7).

In agreement with the low selectivity of the binding site, we observed a difference in the orientation of chelerythrine across two complexes in the asymmetric unit, with a flip along its longer axis (while planar orientation of the ligand is well-defined, flipped orientations within the plane are possible). Chelerythrine is sandwiched between the A14-U55 base pair at the bottom and A56 of the U51-A56-U96 base triple at the top, effectively intercalating between U55 and A56 in a manner that mimics the intercalating base in certain T-loops^7^ (Fig. 1l; Supplementary Fig. S8). In addition, in one copy the positive charge on the nitrogen of the endocyclic quaternary amine facilitates cation-π interaction with the pyrimidine ring of A56. Notably, the ether oxygens of chelerythrine, which tend to be weak H-bond acceptors, do not appear to be utilized for recognition by the ligand binding site and no localized water molecules were observed mediating interactions with the ligand. Additionally, the binding site appears compatible with ligands larger than chelerythrine, consistent with the reported binding of staurosporine (Supplementary Fig. S10)^3^.

The proflavine intercalates between U55 and A56 similarly to chelerythrine (Supplementary Fig. S9). The presence of exocyclic amine groups in proflavine allows H-bonds with phosphate groups of U55 and A56. Moreover, proflavine (p*K*_a_ = 9.4^8^) is expected to be protonated under our crystallization conditions (pH 7.5), enabling a cation–π interaction with the pyrimidine ring of A56, analogous to chelerythrine. Nevertheless, the data preclude unambiguous determination of the ligand orientation in the pocket. Possibly the observed electron density results from a mixture of different proflavine orientations around its longer axis, consistent with low selectivity of the binding pocket.

In this work, we use antibody-assisted RNA crystallography to determine crystal structures of *yjdF* riboswitch from *R. gauvreauii* in complex with chelerythrine and proflavine. The structures explain the ligand binding promiscuity that distinguishes the *yjdF* riboswitch aptamer from other riboswitches. Both ligands bind primarily via stacking interactions that confer high affinity but low selectivity. The electron-rich internal sides of the pocket may further facilitate binding by interacting with the quadrupole edges of the aromatic rings of the ligand and help accommodate ligands that possess H-bond donors. Thus, the high conservation of nucleotides in the ligand binding pocket appears to be aimed towards the formation of a stable binding site and facilitating conformational changes rather than precise ligand recognition.

Recent work proposed a structural mimicry between *yjdF* aptamer domain and tRNA, highlighting the importance of L2b and L4 and their potential interaction reminiscent of the T–D loop interaction in tRNAs^2^. In our structures, the P1, P3, and P4 helices adopt relative positions in space similar to the acceptor stem, anticodon arm and TψC-arm, respectively, in tRNA-Phe. Although we do not observe any direct interactions between L2b and L4 in the ligand-bound *yjdF* riboswitch from *R. gauvreauii*, L2b and L4 contribute significantly to the riboswitch architecture.

The non-canonical interactions between J3/4 and J4/1 at the base of the P4 stem suggest the potential for stem unwinding in the absence of ligand to allow interaction with the downstream RBS. These regions become more structured upon ligand binding in in-line probing experiments^3^. Based on these observations, we propose a model wherein the P4 stem serves as a thermodynamically unstable transducer capable of sequestration or release of the RBS depending on the conformation of the aptamer domain. The event of ligand binding triggers organization of J3/4 and J4/1 and the pairing interactions at the base of P4. This extended pairing stabilizes the P4 stem-loop, partially sequesters residues 101–110 and favors a conformation unfavorable for pseudoknot formation with RBS.

Taken together, our study provides a structural basis for low ligand selectivity of the *yjdF* riboswitch and suggests a potential mechanism through which the *yjdF* aptamer domain could convert ligand binding into changes in RBS accessibility. Our structures lay a foundation for studies into how the *yjdF* riboswitch controls gene expression.

### Supplementary information

Supplementary information, Figures and Table

## Data Availability

Atomic coordinates and structure factors for the reported crystal structures have been deposited at the PDB under accession codes 8UIW and 8UTA for the structures of *yjdF/*chelerythrine and *yjdF/*proflavine, respectively.

## References

[1] Weinberg Z (2010). Genome Biol..

[2] Trachman RJ (2022). J. Biol. Chem..

[3] Li S (2016). RNA.

[4] Madoori PK (2009). EMBO J..

[5] Koldobskaya Y (2011). Nat. Struct. Mol. Biol..

[6] Gong, S. et al. *J. Theor. Biol.* **439**, 152–159 (2018). 10.1016/j.jtbi.2017.12.007 29223402

[7] Chan CW (2013). Wiley Interdiscip. Rev. RNA.

[8] Kemp S (2007). Supramol. Chem..

[9] Leontis NB, Westhof E (2001). RNA.

