# Supplementary material for: Structural basis for promiscuity in ligand recognition by yjdF riboswitch
Source: Cell Discov. 2024 Apr 2;10:37. doi: 10.1038/s41421-024-00663-2 (PMC10987639; doi:10.1038/s41421-024-00663-2)
Supplement: Supplementary file 1 — Supplementary information, Figures and Table [file 41421_2024_663_MOESM1_ESM.pdf]

Supplementary Information

Structural basis for promiscuity in ligand recognition by yjdF riboswitch

Daniel Krochmal[1], Christina Roman[1], Anna Lewicka[1], Yaming Shao[1], Joseph A. Piccirilli[1,2]*

*email: jpicciri@uchicago.edu

[1]*Department of Biochemistry and Molecular Biology, The University of Chicago, Chicago, IL, USA*

[2]*Department of Chemistry, The University of Chicago, Chicago, IL, USA*

Materials and Methods

Supplementary Note S1

Supplementary Figures S1 – S11

Supplementary Table S1

Supplementary References

MATERIALS AND METHODS

RNA synthesis and purification

DNA templates for transcription reactions were prepared by PCR amplification of ssDNA oligomer with T7 promoter sequence purchased from Integrated DNA Technologies (IDT, www.idtdna.com). The first two nucleotides of the reverse primer contained 2′-OMe modifications to reduce transcriptional heterogeneity at the 3′ end[1]. RNA was prepared by *in vitro* transcription for 3 hours at 37°C in buffer containing 40 mM Tris-HCl pH 8.0 (measured at 25°C), 2 mM spermidine, 10 mM NaCl, 25 mM $MgCl_2$, 10 mM DTT, 40 U/ml murine RNase Inhibitor (New England Biolabs), 5 U/ml TIPPase (New England Biolabs), 5 mM of each NTPs, DNA template 100 pmol/ml, 50 µg/ml homemade T7 RNA polymerase[2]. Transcription reactions were quenched by adding 5 U/ml RNase free DNase I (Promega) and incubating at 37°C for 30 minutes. Following DNase treatment, the RNA was purified by denaturing polyacrylamide gel electrophoresis. The corresponding RNA band was visualized by UV shadowing and excised from the gel. RNA was eluted overnight at 4°C in 10 mM Tris pH 8.0 (measured at 25°C), 2 mM EDTA, 300 mM NaCl buffer. The buffer for eluted RNA was exchanged 3 times for pure water using 10 kDa-cutoff size-exclusion column (Amicon, www.emdmillipore.com). RNA was collected, aliquoted and stored at -80°C until further use.

Crystallization

An aliquot of RNA sample was refolded in 50 mM Tris-HCl pH 7.5 (measured at 25°C), 100 mM KCl, 10 mM $MgCl_2$. For refolding, RNA was heated at 90°C for 1 minute in water, added appropriate volume of 10x folding buffer and then incubated at 50°C for 10 minutes in 1x folding buffer followed by incubation at room temperature for 10 minutes. The refolded RNA was then incubated with 2 molar equivalents of chelerythrine chloride (Tocris, www.tocris.com) or proflavine hemisulfate (Sigma-Aldrich, www.sigmaaldrich.com) at room temperature for 30 min, followed by 30 min incubation with 1.1 molar equivalents of the Fab BL3-6 with S97N mutation in the light chain[3]. The complex was then concentrated to 17 mg/ml using 10 kDa-cutoff, Amicon Ultra-15 column (www.emdmillipore.com). The formation of Fab–RNA complex was confirmed by native polyacrylamide gel electrophoresis (nPAGE). To decrease the number of nucleation events, Fab-RNA complexes were then passed through 0.2 µm cutoff, Millipore centrifugal filter units (www.emdmillipore.com). A Mosquito liquid handling robot (SPT Labtech, www.sptlabtech.com) was used to set up high-throughput 100 nl + 100 nl hanging-drop vapor-diffusion crystallization screens at room temperature using commercially available screening kits from Hampton Research and Jena Bioscience. Crystals appeared and grew to full size within two weeks at 22°C

in a drop with well condition 0.1 M HEPES pH 7.5, 0.2 M ammonium acetate, 25% PEG 3350. For SAD phasing, the crystals were then transferred to a solution containing 0.1 M HEPES pH 7.5, 0.2 M ammonium acetate, 25% PEG 3350, 10 mM potassium osmate(VI) and incubated at 22°C for 24h followed by rinsing with the mother liquor. Crystals were immediately flash-frozen in liquid nitrogen after being fished out from the drops and taken to Argonne National Laboratory for collecting the X-ray diffraction data.

Structural data collection, processing and analysis

The X-ray diffraction data sets were collected at the Advanced Photon Source NE-CAT section beamlines 24-ID-C and 24-ID-E for chelerythrine and proflavine complexes, respectively. The anomalous data from the chelerythrine complex-derived crystals were collected at 1 Å wavelength. All the datasets were then integrated and scaled using its on-site RAPD automated programs (https://rapd.nec.aps.anl.gov/rapd/). Initial phases were obtained by molecular replacement (MR) with previously reported structure of Fab BL3-6 (PDB code: 7SZU)[4] as the search model using Phaser on Phenix . To complete the building of the RNA, further phasing of the chelerythrine complex dataset was performed via osmium SAD approach using AutoSol pipeline with the partial solution from MR. Iterative model building and restrained refinement was performed using COOT[5], and phenix.refine[6]. RNA was built unambiguously by modeling the individual nucleotides into the electron density map obtained from the MR-SAD or MR for chelerythrine or proflavine complex, respectively. The nucleotides at positions 76-81 (P3 stem-loop) in ASU Copy 2 were omitted in model building due to poor quality of the electron density map in this region. Water molecules were added manually for the positive electron density in the map based on their possibility to form hydrogen bonds with protein or RNA residues. All structure related figures were made in PyMOL (Schrodinger, www.pymol.org) and figure labels were edited in CorelDraw (Corel Corporation, http://www.corel.com).

Supplementary Note S1

Crystal contacts mediated by the P4 stem-loop: The Fab-RNA complexes of *yjdF* riboswitch bound to chelerythrine and proflavine crystallized in P1 space group and contain two copies of the complex per asymmetric unit. Notably, corresponding copies in both complexes exhibit a similar fold **(Supplementary Fig. S2; RMSD = 1.528 Å** between copy 1 from *yjdF*/chelerythrine and *yjdF*/proflavine complex**)**. However, significant difference is observed between the two copies within the asymmetric unit for both complexes (**Supplementary Fig. S2; RMSD = 4.454 Å and 4.226 Å** for chelerythrine and proflavine complex, respectively). This increased RMSD arises partially from the varying angle at which the P4 protrudes from the central core, as excluding P4 from RMSD calculation reduces the discrepancy. The remaining difference in RMSD may be explained by a slight variation in angles at which the remaining stems protrude from the central core.

Further examination reveals differences in crystal contacts, particularly in the interactions involving P4 **(Supplementary Fig. S11)**; P4 stem-loop from copy 2 (referred to as $P4_2$) forms extensive interactions with the $P3_1$ stem-loop (referred to as $P3_{1-1}$) and the base of the $P1_1$ stem from another symmetry mate ($P1_{1-2}$), contributing nucleotides to the helical arrangement in both instances. Specifically, C106 from $P4_2$ intercalates between G74 and G75 from $P3_{1-1}$ and forms a WCF base pair with G82 from $P3_{1-1}$ that stacks upon the G74-C83 WCF base pair. Meanwhile, C107 from $P4_2$ forms a WCF base pair with the overhanging G1, that effectively extends the helical stack of $P1_{1-2}$.

In contrast, these interactions are entirely absent in $P4_{1.}$ Instead, $P4_1$ forms a network of interactions with the light chain variable domain of Fab BL3-6, mainly via the phosphoribose backbone **(Supplementary Fig. S11)**. Specifically, S31 and S32 from CDR-L1 form hydrogen bonds with OP1 of U108 and 2'OH of U109, respectively. S68 and G69 from Fab's light chain framework region 3 (FR3) stabilize the phosphoribose backbone of G111 and A110, while R67 from FR3 utilizes its NE and NH2 groups to hydrogen bond with 2'OH of A110 and 3'O of C107, respectively. Lastly, S53 from CDR-L2 interacts with O2 of U109.

The network of interactions formed by the P4 stem-loops from the two copies likely contributes to the differences in the protrusion angle of P4 from the central core. Nonetheless, this variation in the protrusion angle of P4 implies that there must be conformational flexibility in this region to allow for such differences.

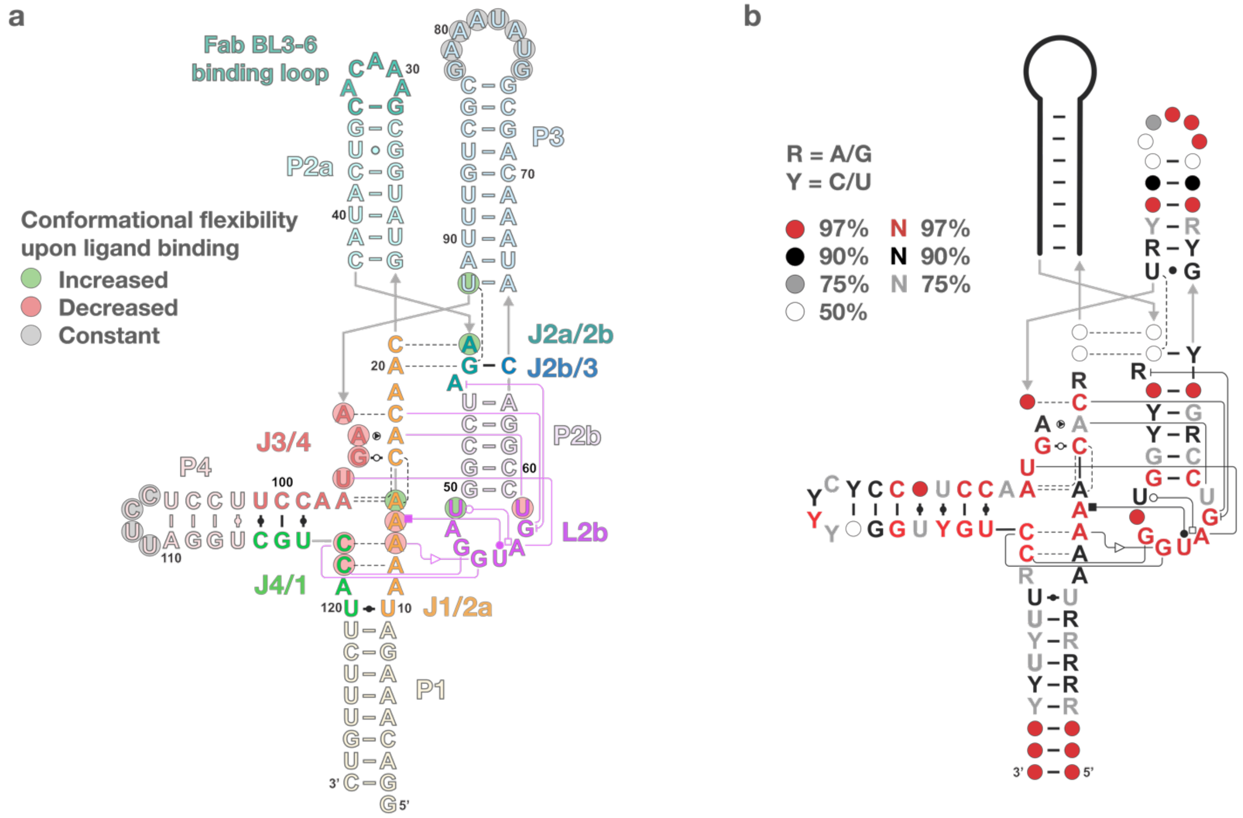

Supplementary Fig. S1 **a** In-line probing and **b** phylogenetic conservation data[7] mapped on the crystal-derived secondary structure diagram of the *yjdF* riboswitch. The secondary and tertiary interactions are denoted using the Westhof-Leontis notation[8], with the L2b-mediated interactions colored magenta. Dashed lines indicate interactions mediated by 2'OH of ribose.

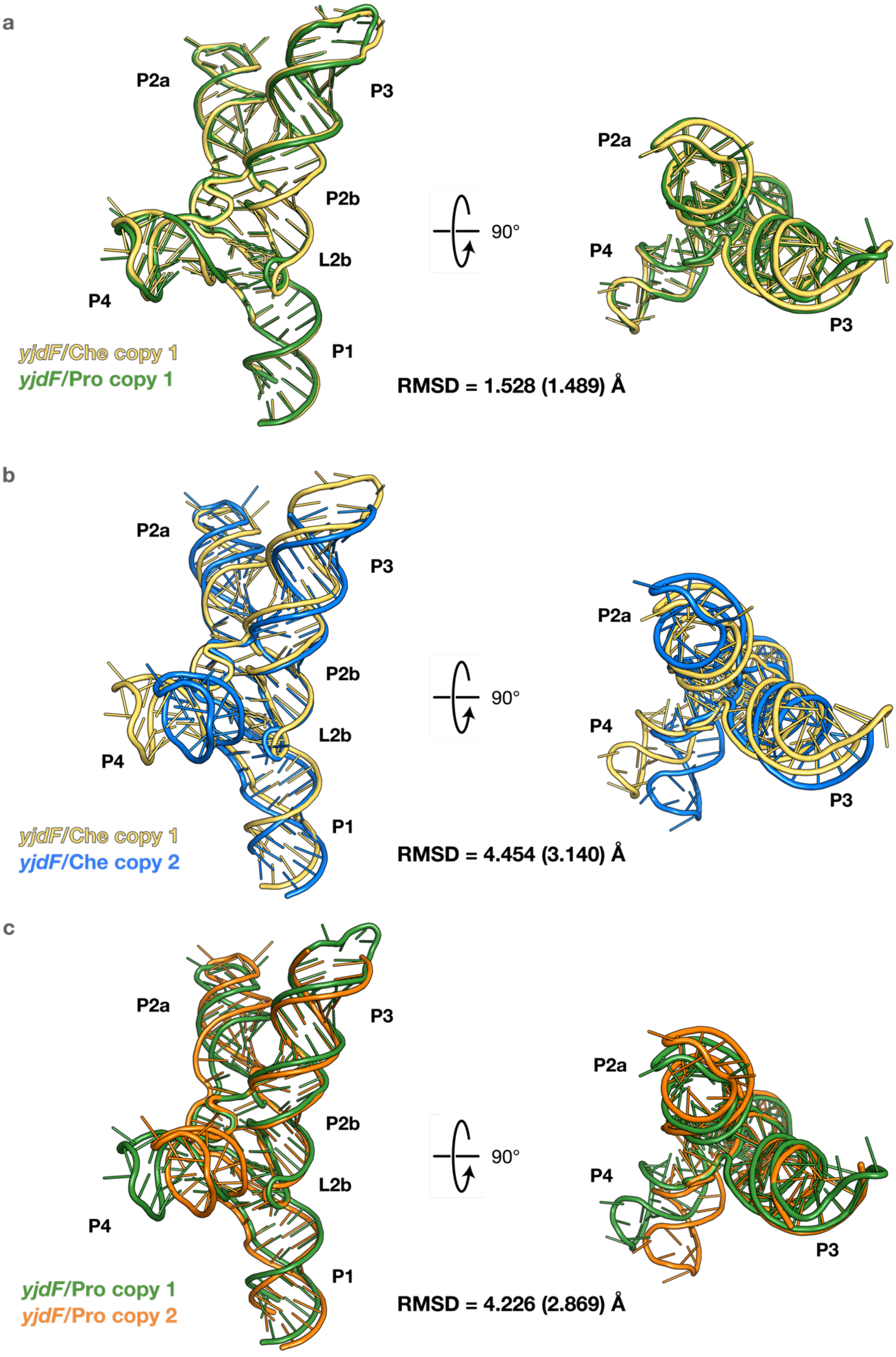
a
P2a
P3
P2b
L2b
P4
P1
90°
P2a
P4
P3
yjdF/Che copy 1
yjdF/Pro copy 1
RMSD = 1.528 (1.489) Å
b
P2a
P3
P2b
L2b
P4
P1
90°
P2a
P4
P3
yjdF/Che copy 1
yjdF/Che copy 2
RMSD = 4.454 (3.140) Å
c
P2a
P3
P2b
L2b
P4
P1
90°
P2a
P4
P3
yjdF/Pro copy 1
yjdF/Pro copy 2
RMSD = 4.226 (2.869) Å

Supplementary Fig. S2 Comparison of global architectures of *yjdF R. gauvreauii* aptamer domain. Superposition of **a** *yjdF*/chelerythrine and *yjdF*/proflavine complex. **b** both copies of *yjdF*/chelerythrine complex from the same asymmetric unit. **c** both copies of *yjdF*/proflavine complex from the same asymmetric unit. The values in parentheses indicate the RMSD calculated after excluding P4. All figures are colored analogously for facile comparison.

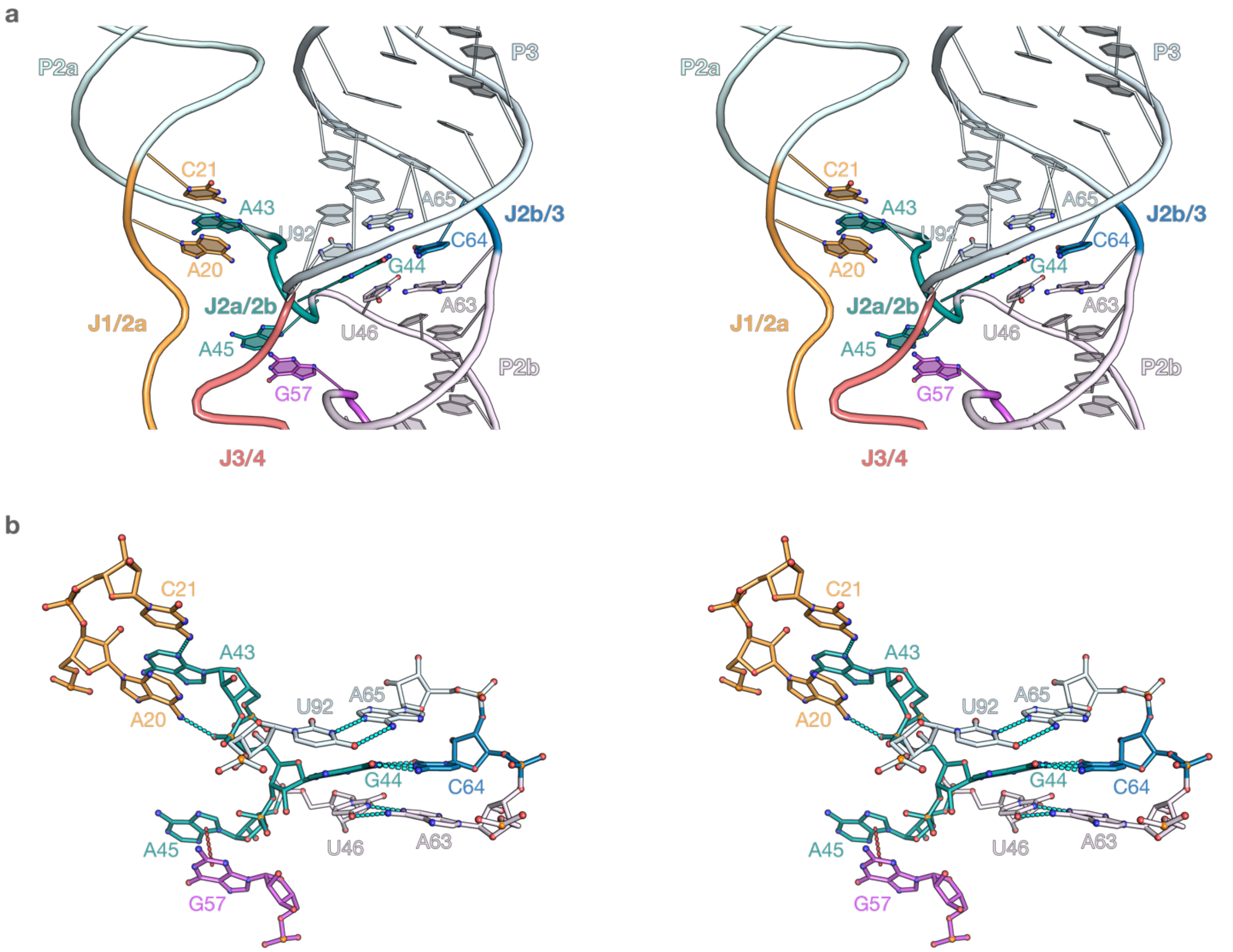

Supplementary Fig. S3 Interactions between J2a/2b and J2b/3 facilitate the coaxial arrangement between P2b and P3. Parallel-eye stereoscopic images are presented. Cyan dashes indicate heteroatoms within hydrogen-bonding distance (3.5 Å). Red dashes indicate π-π stacking of aromatic rings.

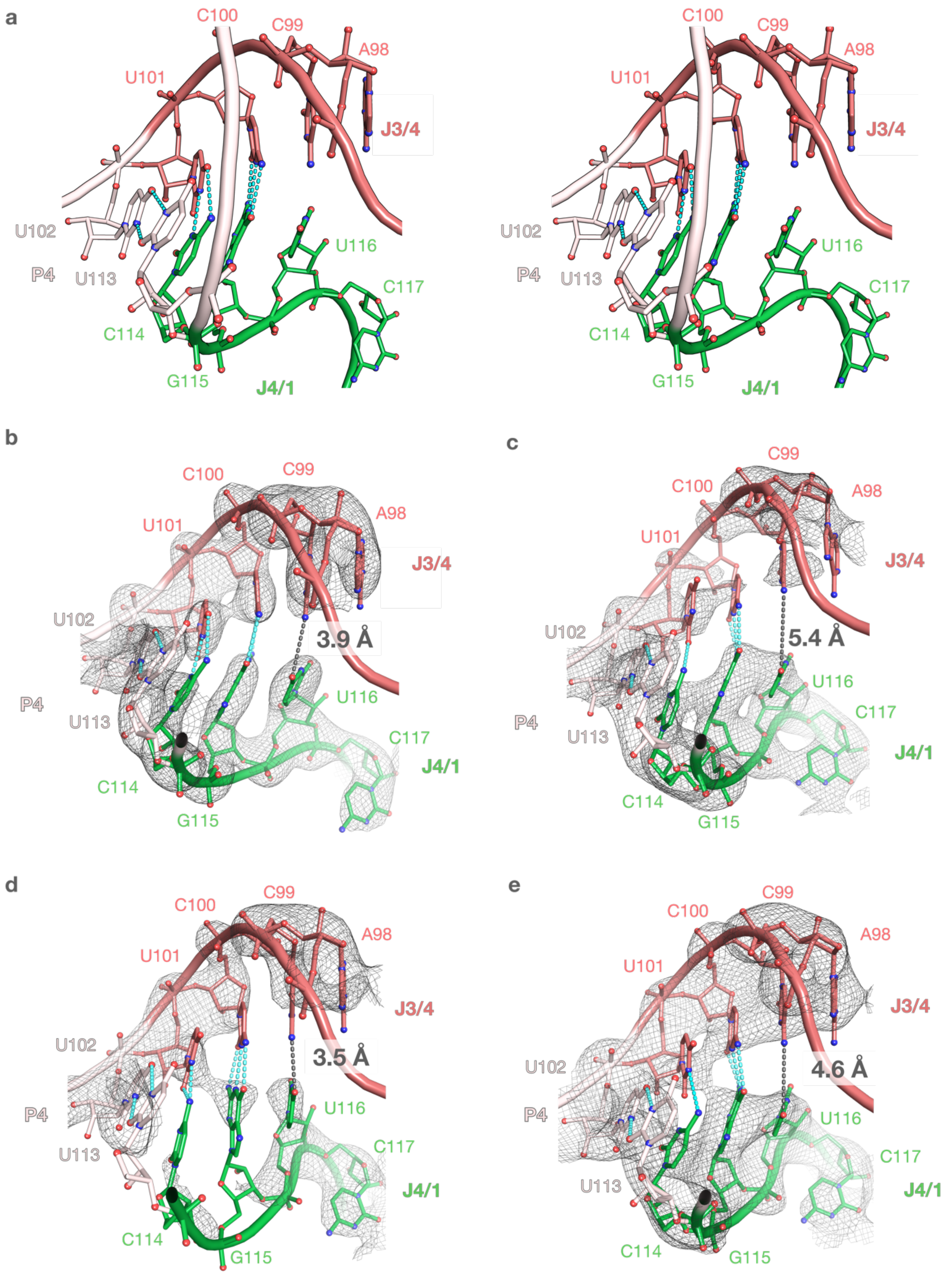
a
C100
C99
A98
U101
J3/4
U102
P4
U113
U116
C117
C114
G115
J4/1
b
C100
C99
A98
U101
J3/4
U102
3.9 Å
P4
U113
U116
C117
J4/1
C114
G115
c
C99
C100
A98
U101
J3/4
U102
5.4 Å
U116
P4
C117
U113
J4/1
C114
G115
d
C99
C100
A98
U101
J3/4
U102
3.5 Å
U116
P4
U113
C117
J4/1
C114
G115
e
C99
C100
A98
U101
J3/4
U102
4.6 Å
P4
U116
C117
U113
J4/1
C114
G115

Supplementary Fig. S4 Conformational heterogeneity of J3/4 and J4/1 in the vicinity of P4 demonstrated by interactions between nucleotides of J3/4 and J4/1. **a** Parallel-eye stereoscopic image of the interactions between J3/4 and J4/1 at the base of P4 stem from copy 1 of *yjdF*/chelerythrine complex. Interactions between J3/4 and J4/1 from **b, c** copies 1 and 2 of *yjdF*/chelerythrine complex, respectively, and **d, e** copies 1 and 2 of *yjdF*/proflavine complex, respectively. Cyan dashes indicate heteroatoms within hydrogen-bonding distance (3.5 Å). The distance between N4 of C99 and O4 of U116 is indicated with dark grey dashes. Meshes represent the $2|F_o| - |F_c|$ electron density map at 1.5σ contour level and carve radius 2.5 Å. All figures are colored analogously for facile comparison.

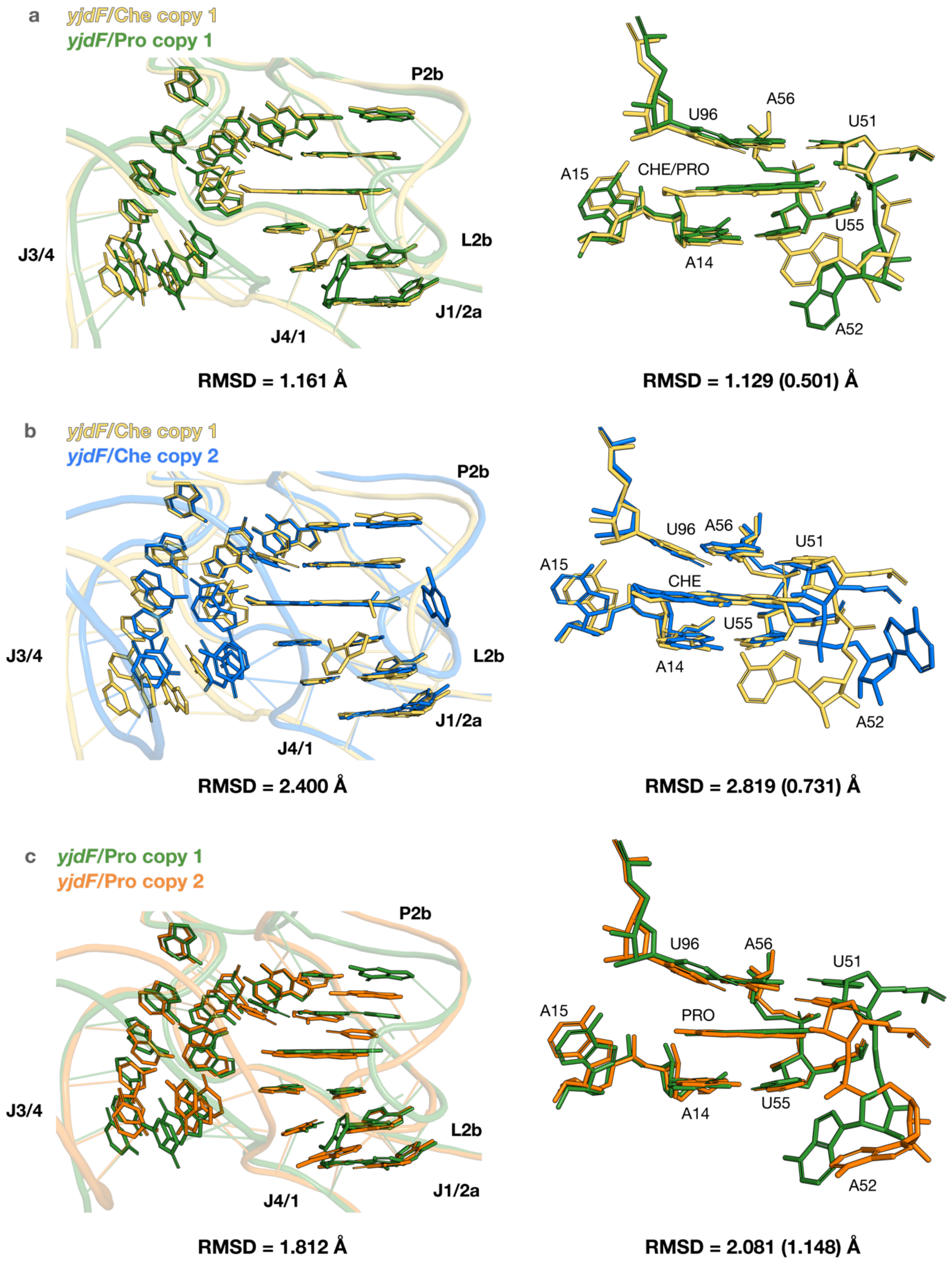
a
yjdF/Che copy 1
yjdF/Pro copy 1
P2b
J3/4
L2b
J1/2a
J4/1
U96
A56
U51
A15
CHE/PRO
U55
A14
A52
RMSD = 1.161 Å
RMSD = 1.129 (0.501) Å
b
yjdF/Che copy 1
yjdF/Che copy 2
P2b
J3/4
L2b
J1/2a
J4/1
U96
A56
U51
A15
CHE
U55
A14
A52
RMSD = 2.400 Å
RMSD = 2.819 (0.731) Å
c
yjdF/Pro copy 1
yjdF/Pro copy 2
P2b
J3/4
L2b
J1/2a
J4/1
U96
A56
U51
A15
PRO
A14
U55
A52
RMSD = 1.812 Å
RMSD = 2.081 (1.148) Å

Supplementary Fig. S5 Comparison of the ligand binding pockets from *yjdF R. gauvreauii* aptamer domain. Superposition of the central core and the ligand binding site from **a** *yjdF*/chelerythrine and *yjdF*/proflavine complex. **b** both copies of *yjdF*/chelerythrine complex from the same asymmetric unit. **c** both copies of *yjdF*/proflavine complex from the same asymmetric unit. The values in parentheses indicate the RMSD calculated after excluding A52. All figures are colored analogously for facile comparison.

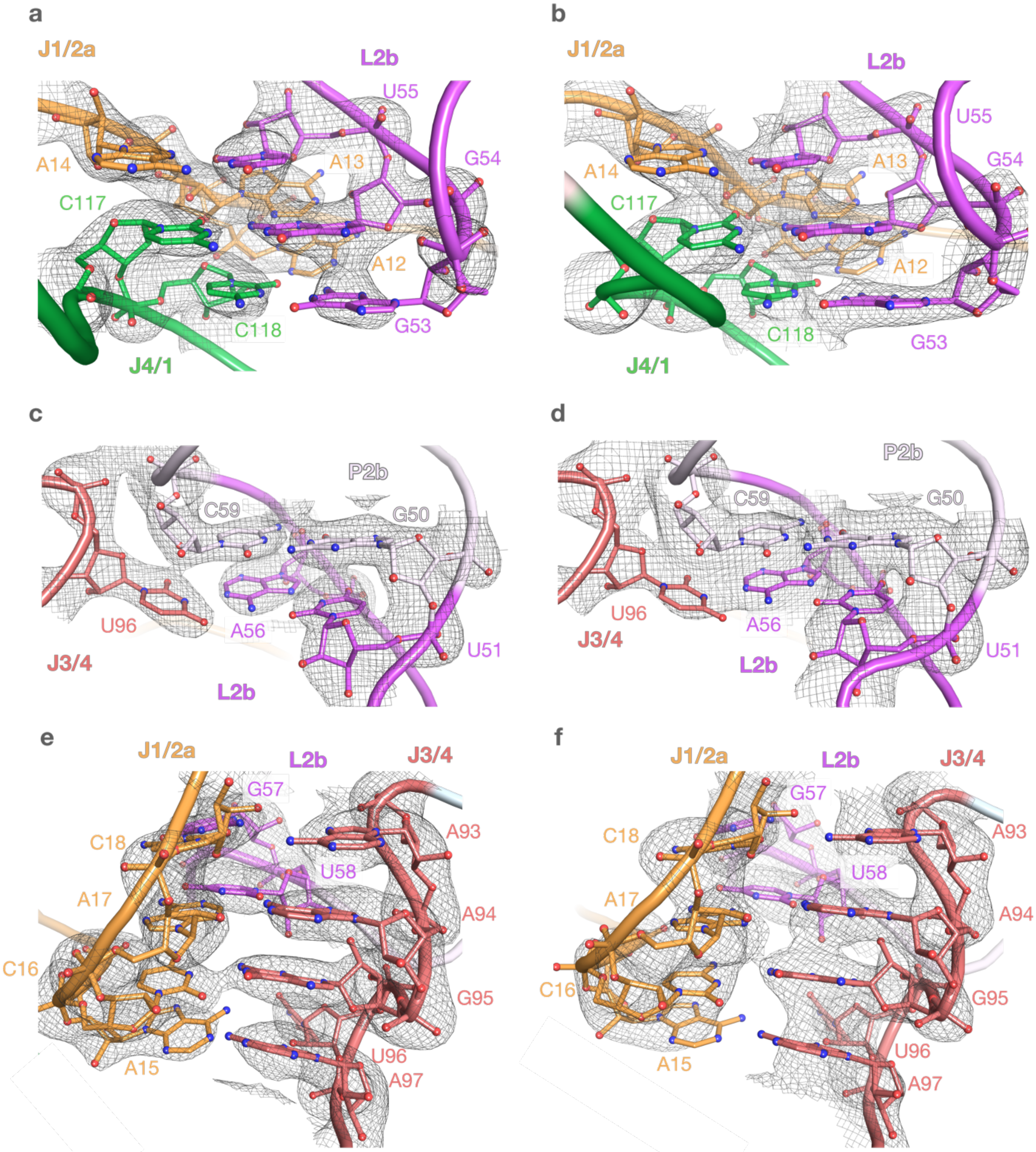

Supplementary Fig. S6 The structure of the central core of **a, c, e** *yjdF*/chelerythrine complex and **b, d, f** *yjdF*/proflavine complex with the view on the **a, b** bottom, **c, d** top and **e, f** side of the ligand-binding pocket. Meshes represent the $2|F_o| - |F_c|$ electron density map at 1.5σ contour level and carve radius 2.5 Å. All figures are colored analogously for facile comparison.

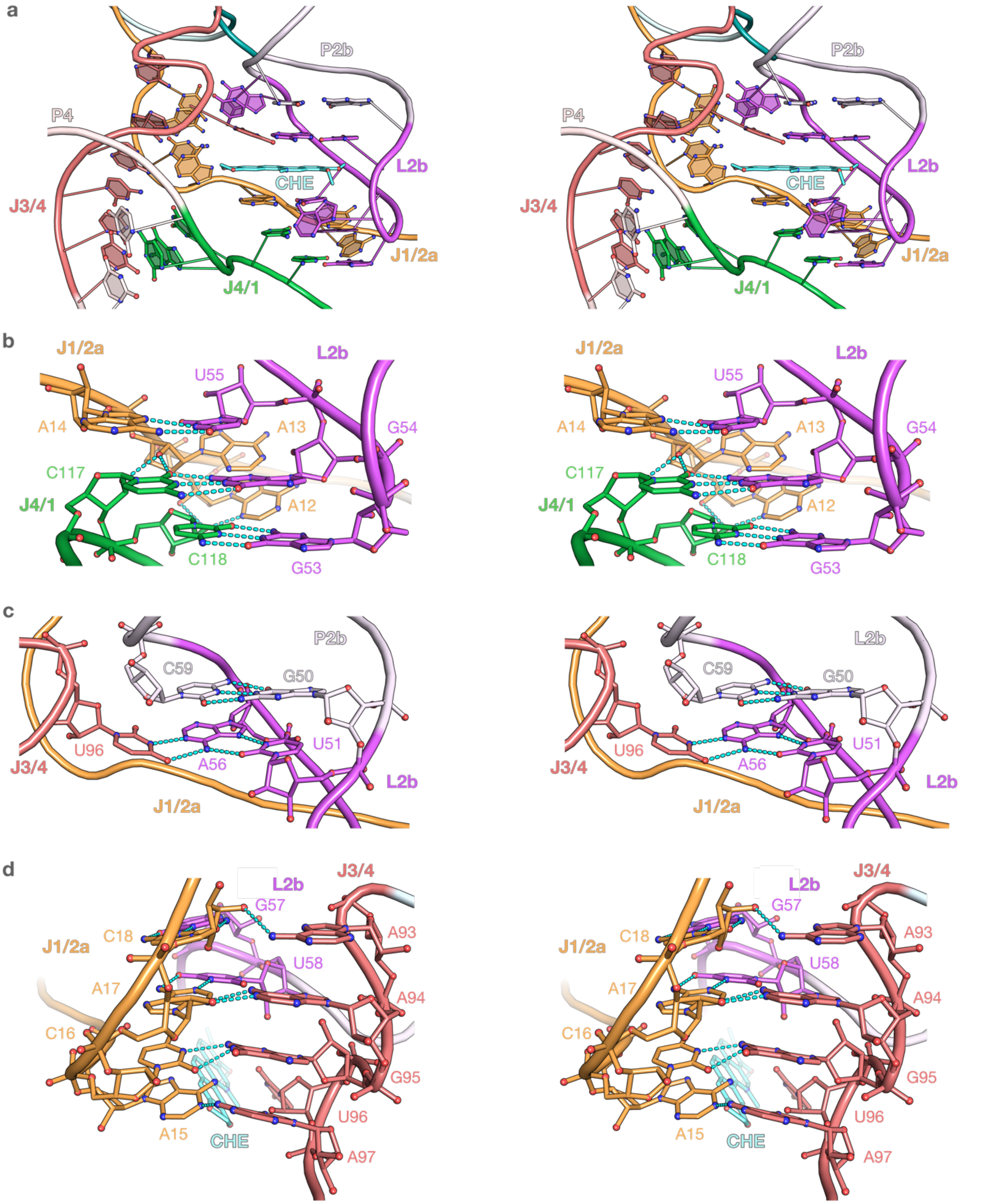

Supplementary Fig. S7 Parallel-eye stereoscopic images of the central core of *yjdF* riboswitch bound to chelerythrine. **a** Overview of the central core of *yjdF* riboswitch with the ligand-binding site. Detailed interactions forming the **b** bottom, **c** top and **d** side of the ligand-binding pocket. Cyan dashes indicate heteroatoms within hydrogen-bonding distance (3.5 Å).

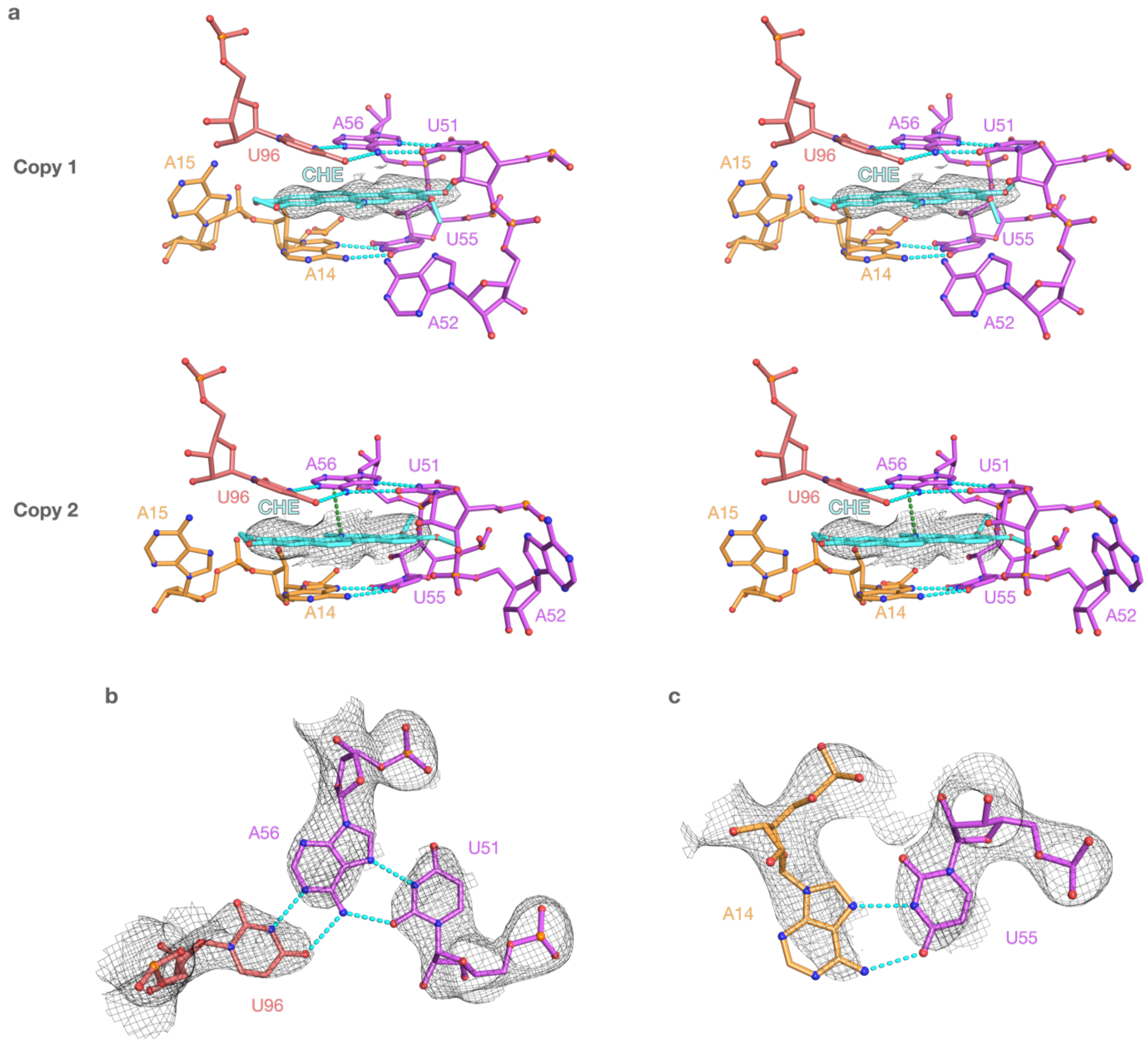

Supplementary Fig. S8 The architecture of the ligand-binding site of *yjdF*/chelerythrine complex. **a** Parallel-eye stereoscopic images of the ligand-binding site from both copies of the *yjdF* riboswitch/chelerythrine complex. Meshes represent the composite omit map at 1.5σ contour level and carve radius 1.8 Å. **b, c** The platform for stacking with the top and bottom face of the ligand, respectively. Meshes represent the $2|F_o| - |F_c|$ electron density map at 1.5σ contour level and carve radius 2.5 Å. Cyan dashes indicate heteroatoms within hydrogen-bonding distance (3.5 Å). Green dashes indicate interactions mediated by the ligand.

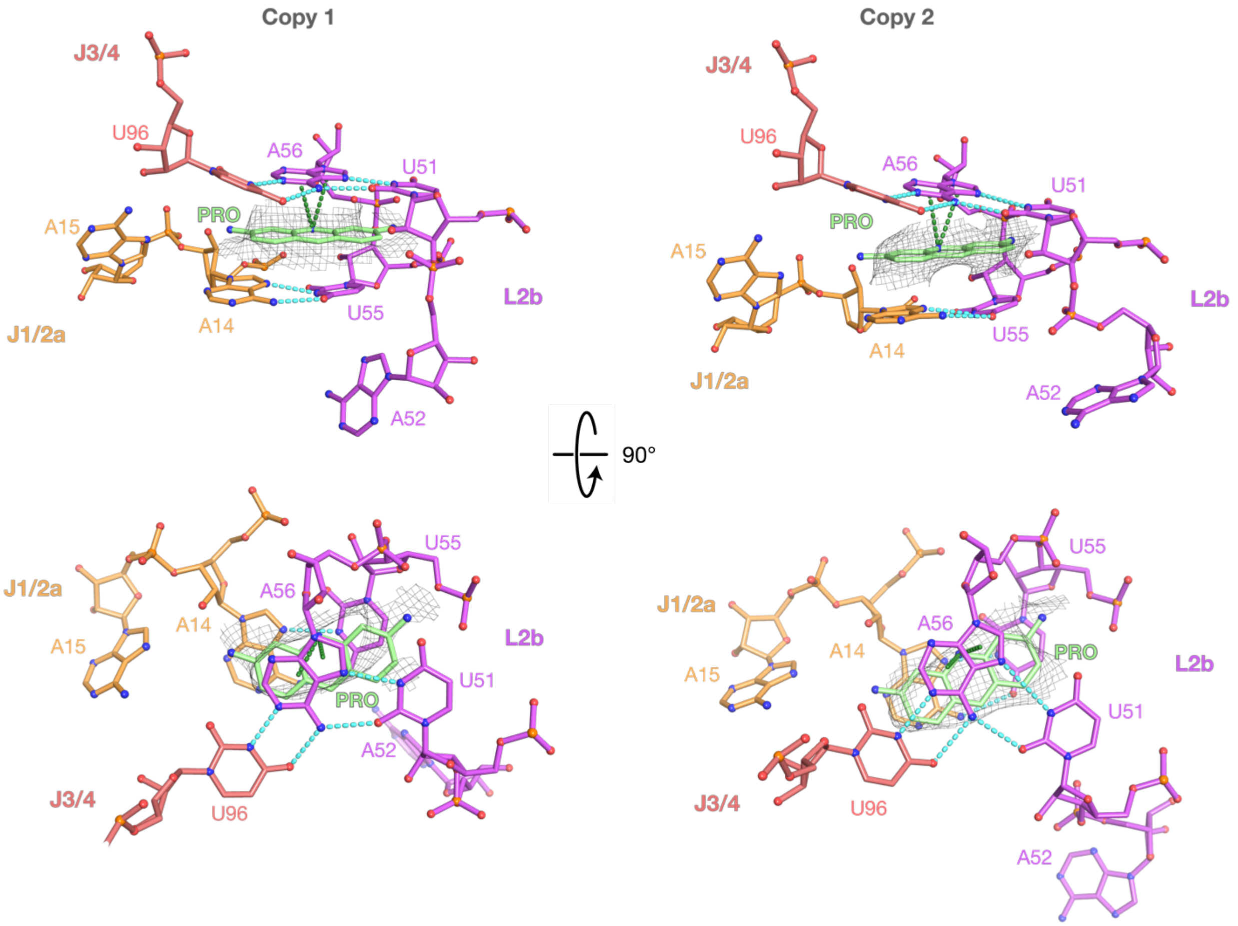

Supplementary Fig. S9 The architecture of the ligand-binding site of *yjdF*/proflavine complex demonstrating the nucleotides in the direct proximity of the ligand. Cyan dashes indicate heteroatoms within hydrogen-bonding distance (3.5 Å). Green dashes indicate interactions mediated by the ligand. Meshes represent the composite omit map at 1σ contour level and carve radius 1.8 Å. All figures are colored analogously for facile comparison.

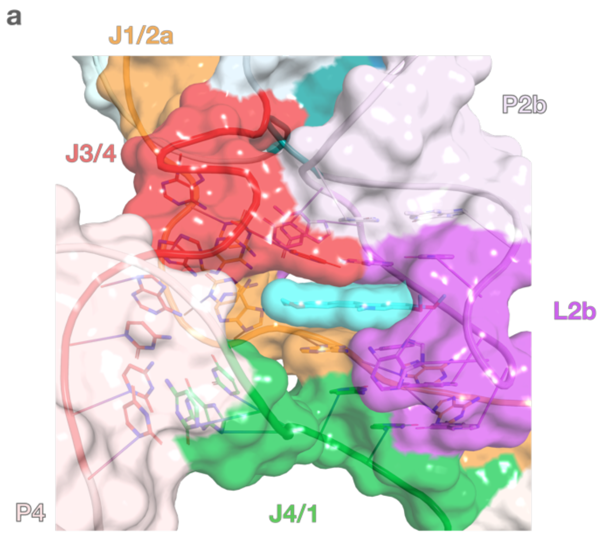

Supplementary Fig. S10 Molecular surface of **a** *yjdF*/chelerythrine and **b** *yjdF*/proflavine complex demonstrating the size of the ligand-binding pore. All figures are colored analogously for facile comparison.

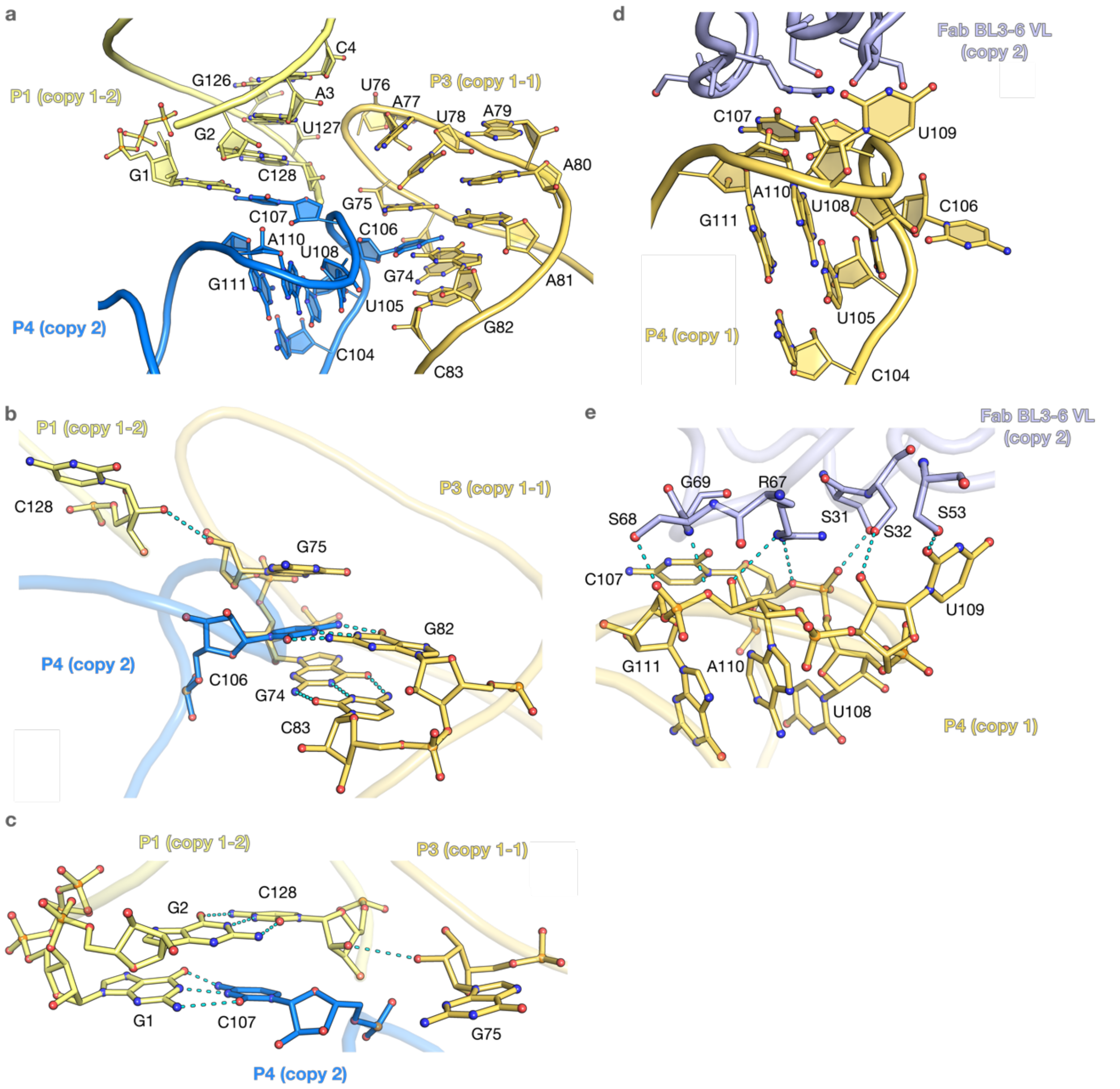

Supplementary Fig. S11 Crystal contacts mediated by the P4 stem-loop in *yjdF*/chelerythrine complex. **a** Overview of the crystal contacts mediated by P4 from copy 2. U109 from P4 copy 2 is omitted for clarity. **b, c** Crystal contacts mediated by C106 and C107, respectively, from copy 1 belonging to two symmetry mates (referred to as copy 1-1 and copy 1-2). **d** Overview and **e** detailed crystal contacts mediated by P4 from copy 1. Fab BL3-6 VL corresponds to the variable domain of Fab BL3-6 light chain.

Supplementary Table S1 Data collection and refinement statistics.

	yjdF/Che	*yjdF*/Pro
**Wavelength**	1.0003	0.9792
**Resolution range**	77.3 - 2.77 (2.869 - 2.77)	63.79 - 3.055 (3.164 - 3.055)
**Space group**	P 1	P 1
**Unit cell**	65.792 77.438 95.848 87.19 88.426 88.046	63.873 75.138 95.727 87.25 87.987 87.719
**Total reflections**	254954 (24404)	149070 (14031)
**Unique reflections**	45080 (4337)	32521 (2283)
**Multiplicity**	5.7 (5.4)	4.6 (4.4)
**Completeness (%)**	93.31 (89.07)	93.17 (68.02)
**Mean I/sigma(I)**	18.88 (0.83)	8.57 (0.48)
**Wilson B-factor**	99.15	121.62
R_{merge}	0.05912 (1.64)	0.1034 (2.688)
R_{meas}	0.06502 (1.814)	0.1171 (3.056)
R_{pim}	0.02695 (0.7683)	0.05418 (1.432)
$CC_{1/2}$	0.992 (0.542)	0.995 (0.34)
**CC***	0.998 (0.839)	0.999 (0.712)
CC_{anom}	0.657 (-0.025)	-
**Reflections used in refinement**	44804 (4334)	31352 (2282)
**Reflections used for R-free**	1998 (187)	2003 (139)
R_{work}	0.2112 (0.5772)	0.2244 (0.4328)
R_{free}	0.2308 (0.5970)	0.2694 (0.4349)
CC_{work}	0.942 (0.636)	0.982 (0.566)
CC_{free}	0.977 (0.577)	0.973 (0.368)
**RMS(bonds)**	0.008	0.008
**RMS(angles)**	1.34	1.20
**Ramachandran favored (%)**	99.20	99.20
**Ramachandran allowed (%)**	0.80	0.80
**Ramachandran outliers (%)**	0.00	0.00
**Rotamer outliers (%)**	1.33	0.00
**Clashscore**	8.66	8.63
**Average B-factor**	131.07	167.08

SUPPLEMENTARY REFERENCES

1. Kao, C., Rüdisser, S. & Zheng, M. *Methods* **23**, 201–205 (2001).

2. Rio, D. C. *Cold Spring Harb. Protoc.* **2013**, (2013).

3. Koirala, D. et al. *Nucleic Acids Res.* **46**, 2624–2635 (2018).

4. Rees, H. C., Gogacz, W., Li, N.-S., Koirala, D. & Piccirilli, J. A. *ACS Chem. Biol.* (2022).

5. Emsley, P., Lohkamp, B., Scott, W. G. & Cowtan, K. *Acta Crystallogr. D Biol. Crystallogr.* **66**, 486–501 (2010).

6. Adams, P. D. et al. *Acta Crystallogr. D Biol. Crystallogr.* **66**, 213–221 (2010).

7. Li, S., Hwang, X. Y., Stav, S. & Breaker, R. R. *RNA* **22**, 530–541 (2016).

8. Leontis, N. B. & Westhof, E. *RNA* **7**, 499–512 (2001).
